# Prospective association between psychopathological symptoms in childhood and asthma in adolescence: Results from the GINIplus and LISA birth cohort studies

**DOI:** 10.1111/pai.70151

**Published:** 2025-07-24

**Authors:** Pia‐Marie Keim, Ellen Greimel, Lisa Feldmann, Charlotte Elisabeth Piechaczek, Carla P. Harris, Claudia Flexeder, Dietrich Berdel, Andrea von Berg, Sibylle Koletzko, Carl‐Peter Bauer, Tamara Schikowski, Gunda Herberth, Joachim Heinrich, Gerd Schulte‐Körne, Marie Standl

**Affiliations:** ^1^ Department of Child and Adolescent Psychiatry, Psychosomatics and Psychotherapy LMU University Hospital, Ludwig‐Maximilians‐University (LMU) Munich Munich Germany; ^2^ German Center for Mental Health (DZPG), Partner Site Munich‐Augsburg Munich Germany; ^3^ Department of Pediatrics Dr. von Hauner Children's Hospital, University Hospital, LMU Munich Munich Germany; ^4^ Institute of Epidemiology, Helmholtz Zentrum München—German Research Centre for Environmental Health Neuherberg Germany; ^5^ Institute and Clinic for Occupational, Social and Environmental Medicine University Hospital, Ludwig Maximilians‐Universität München Munich Germany; ^6^ German Center for Lung Research (DZL) Munich Germany; ^7^ Research Institute, Department of Pediatrics Marien‐Hospital Wesel Wesel Germany; ^8^ Department of Pediatrics, Gastroenterology and Nutrition School of Medicine Collegium Medicum University of Warmia and Mazury Olsztyn Poland; ^9^ Department of Pediatrics Technical University of Munich Munich Germany; ^10^ Department of Epidemiology IUF‐Leibniz Research Institute for Environmental Medicine Düsseldorf Germany; ^11^ Department of Environmental Immunology Helmholtz Centre for Environmental Research‐ UFZ Leipzig Germany; ^12^ Allergy and Lung Health Unit, Centre for Epidemiology and Biostatistics, School of Population & Global Health The University of Melbourne Melbourne Victoria Australia; ^13^ German Center for Child and Adolescent Health (DZKJ) Munich Germany

**Keywords:** asthma, epidemiology, psychopathological symptoms, puberty, sex difference, Strengths and Difficulties Questionnaire

## Abstract

**Background:**

Although there is a high co‐occurrence of psychopathological symptoms and asthma, longitudinal studies considering asthma endotypes in childhood and adolescence are scarce. Therefore, this study examined the prospective association between psychopathology in childhood and current atopic and non‐atopic asthma in adolescents, considering sex and puberty‐related characteristics.

**Methods:**

The study includes 3584 participants (1790 females, 1794 males) from two German birth cohort studies, GINIplus and LISA. Psychopathological symptoms at age 10 (*M* = 10.06, SD = .22) were assessed by parent‐reported Strengths and Difficulties Questionnaire (SDQ) and current asthma at age 15 (*M* = 15.07, SD = .30) was defined as a positive parent‐reported medical diagnosis, wheezing, and/or medical treatment against asthma (at least two criteria). Atopic asthma was characterized as current asthma and a positive specific immunoglobulin E (IgE) test (≥0.35 kU/L), non‐atopic asthma as current asthma without sensitization. Data were analyzed by logistic regression and multinomial regression models, controlling for potential covariates and confounders.

**Results:**

Psychopathological symptoms at age 10 were associated with asthma at age 15 (odds ratio (OR) = 1.69, 95% confidence interval (CI) = 1.04–2.92, *p* = .035). When examining the endotypes, this effect was replicated only for non‐atopic asthma at age 15 (relative risk ratio (RRR) = 2.92, 95% CI = 1.13–7.60, *p* = .028), but not for atopic asthma (*p* = .381). Regarding the SDQ subscales, an association between peer problems at age 10 and atopic asthma at age 15 was revealed (RRR = 2.00, 95% CI 1.01–3.97, *p* = .048). Furthermore, significant interaction effects of puberty onset with psychopathological symptoms (*p* = .020), and particularly with peer problems (*p* = .029), were found on atopic asthma.

**Conclusion:**

The findings highlight the importance of early recognition and treatment of psychopathological symptoms to prevent and reduce development, persistence and exacerbation of asthma endotypes, and point to the necessity for further research into hormonal mechanisms linking psychopathology with atopy.

**Key message**

This large‐scale prospective cohort study showed that psychopathological symptoms in childhood are associated with an increased prevalence of asthma, specifically non‐atopic asthma in adolescence. Considering selected psychopathological symptoms, the association was detected for peer problems and atopic asthma, with this relationship being modified by pubertal status.

## 1 INTRODUCTION

Epidemiological research points out that asthma and psychopathological problems show a high co‐occurrence.^1^, ^2^ Both are highly prevalent in childhood and adolescence, with rates for asthma symptoms of about 10%^3^ and psychopathological symptoms even higher, at over 20%.^4^ While during childhood, there is a male predominance regarding psychopathological symptoms,^5^ more females suffer from psychopathological symptoms in adolescence, thereby particularly exhibiting internalizing problems.^6^ Similar patterns emerge for asthma. Males under the age of 10 are more likely to be affected,^7^ but this sex difference decreases with increasing age, with equal distribution in adolescence^8^ and female predominance during adulthood.^9^ Both asthma and psychopathological symptoms each comprise numerous variants with distinct etiologies, impairments, and underlying causes. A common factor linking them is their association with psychosocial stress. Stress is a key contributor to the onset and maintenance of psychopathological symptoms^10^ and exerts an important influence on immune and inflammatory pathways.^11^ For atopic asthma, which originates from allergic sensitization to inhalant and food allergens, this leads to an exacerbation of asthma symptoms. For non‐atopic asthma, it serves as a direct risk factor for disease onset by increasing susceptibility to respiratory infections, which are considered the most common triggering factor.^12^

So far, the directional relationship between asthma and psychopathology has not been explored sufficiently yet. While one prospective study indicates that asthma in adulthood results in an increased risk of mental health problems,^13^ most of the research, both in childhood and adulthood, suggests that psychopathological symptoms, and specifically internalizing problems, impact the development of asthma and also its symptom severity.^14^, ^15^, ^16^, ^17^, ^18^, ^19^ It should be noted that previous longitudinal studies have mainly examined adults.^14^, ^15^, ^16^ In childhood and adolescence, most studies have taken a cross‐sectional approach^1^, ^19^ and large‐scale, longitudinal studies^18^ are rare. Furthermore, the relationship between psychopathology and different asthma endotypes, such as atopic and non‐atopic asthma, has received little attention so far (but see^1^). However, to develop adapted approaches for prevention and treatment and to optimize outcomes in the long term, it is of high relevance to close this research gap with longitudinal studies taking into account asthma endotypes. The investigation of non‐atopic asthma appears to be of particular importance, as its occurrence increases with age,^20^ yet the underlying mechanisms remain insufficiently elucidated.

Therefore, the main aim of the current large‐scale study, based on two harmonized German birth cohorts, was to (1) prospectively investigate the association between the occurrence of psychopathological symptoms in childhood and the prevalence of asthma in adolescence, also taking selected asthma endotypes (atopic vs. non‐atopic asthma) into account. Based on the trends observed in previous research,^14^, ^15^, ^16^, ^17^, ^18^, ^19^ it was hypothesized that children with psychopathological symptoms are more likely to suffer from asthma in adolescence compared to children without psychopathological symptoms. In view of the age‐specific changes in sex distribution of psychopathological symptoms and asthma and the assumed relevance of pubertal influences on these changes, the second aim of the study was to examine the (2) impact of sex and onset of puberty on this prospective association.

## 2 MATERIALS AND METHODS

### 2.1 Study population

The present study is based on data from the 10‐ and 15‐year follow‐ups from the birth cohort studies GINIplus (The German Infant Study on the influence of Nutrition Intervention PLUS environmental and genetic influences on allergy development) and LISA (Influence of lifestyle factors on the development of the immune system and allergies in East and West Germany). The common aim of the harmonized studies is to describe the natural course of common chronic diseases with a focus on allergic diseases in childhood, adolescence, and young adulthood, taking into account potentially moderating genetic, environmental, psychosocial, and lifestyle factors.

GINIplus is a prospective population‐based birth cohort study. For this purpose, a total of 5991 healthy, term‐born infants were recruited in two German regions (Munich, Wesel) between 1995 and 1998.^21^ Participants with a family history of allergic disease were enrolled in the intervention arm investigating the preventive effect of hydrolyzed formulas. All other participants were followed up in the observational arm. 3317 children (55.4%) of the original study population participated in the 10‐year follow‐up. In the 15‐year follow‐up, 3198 participants (53.4%) were included.

LISA is a population‐based birth cohort study, for which a total of 3094 healthy, term‐born infants in the regions of Munich, Wesel, Leipzig, and Bad Honnef were included between 1997 and 1999.^21^ 1761 families (56.9%) participated in the 10‐year follow‐up and 1740 in the 15‐year follow‐up (56.2%).

In addition to obtaining informed consent, both studies were reviewed and approved by local ethics committees (Bavarian General Medical Council, University of Leipzig, Medical Council of North‐Rhine‐Westphalia). More details about the studies have been described elsewhere.^21^

### 2.2 Assessment of asthma

Current asthma was defined as a positive parent‐report on at least two of the following three questions: Ever being doctor diagnosed (based on yearly information asking for medical diagnosis of asthma in 3rd to 15th year of life), whistling or wheezing symptoms in the past 12 months, and medical treatment in the past 12 months (reviewed by a medical doctor). In asthma cases, two times yes was sufficient regardless of whether the third question was answered in the affirmative, in the negative, or in its absence. In participants included in the reference group (no asthma), either two or all three questions had to be answered in the negative, and no missings were allowed. This approach was chosen in order not to exclude asthma cases in the asthma group, but to generate a control group that was as strictly selected as possible.

If current asthma was present, additional data on allergic sensitization assessed with the CAP‐RAST FEIA test (Phadia GmbH, Thermo Fisher, Freiburg, Germany) were used from participants who voluntarily attended the on‐site examination and consented to a blood sample collection. This comprises a screening test for common inhalant (SX1) and food (FX5) allergens. The presence of allergic sensitization was defined as any specific immunoglobulin E (IgE) antibody value of at least 0.35 kU/L.^22^ Participants with a positive specific IgE test and current asthma were labeled atopic. If current asthma was present but no allergic sensitization was found, the participants were classified as non‐atopic.

To consider additional objective measures in asthma classification, we performed sensitivity analyses adjusting for fractional exhaled nitric oxide (FeNO)^23^, ^24^ measurement and a positive spirometric bronchodilation response^25^ (see Appendix S4).

### 2.3 Measurement of psychopathological symptoms

The Strengths and Difficulties Questionnaire (SDQ) was applied to assess psychopathological symptoms.^26^ The questionnaire is a widely used screening instrument and can be completed by parents and teachers as an external assessment or by the child itself from the age of eleven onwards.^27^ In the present study, the questionnaire at the 10‐year follow‐up was completed by a parent.^28^

The questionnaire includes a total of 25 items on psychopathological problems. Each item is rated on a three‐point Likert scale (0 = “not true”, 1 = “somewhat true”, and 2 = “certainly true”). The sum of the total score can be divided into five subscales: emotional problems, conduct problems, hyperactivity/inattention, peer problems and problems in prosocial behavior.^26^ All (sub‐)scales have shown largely acceptable to good reliability (Cronbach's *α* = .58–.82).^29^

Both the SDQ total score and the values of the SDQ subscales in the 10‐year follow‐up were categorized into three levels based on previous recommendations using existing German cut‐offs for parent‐report: “normal”, “borderline”, and “abnormal”.^28^, ^29^ The category “abnormal” corresponds to significantly increased risk of a clinically relevant disorder.^30^ For the following statistical analyses, a dichotomization of the classification of the characteristics into “borderline/abnormal” versus “normal” was carried out.^28^

### 2.4 Covariates and Confounders

The following analyses were performed by accounting for several covariates (based on the study design) and potential confounders at the time of the outcome assessment, which were determined a priori. According to the study design, all analyses were adjusted for exact age when filling out the test battery at the 15‐year follow‐up, sex, study group (GINIplus observation arm/GINIplus intervention arm/LISA), and recruitment region (Munich/Leipzig/Bad Honnef/Wesel). Socioeconomic status (SES) of the respective household was represented by parental education level, measured by the highest grade completed by either the mother or the father based on the German educational system (low < 10th grade/medium = 10th grade/high > 10th grade). Parental atopy was defined as at least one parent suffering from an atopic disease at the time of the child's birth (yes/no). Additionally, information on early‐life infections, specifically lower respiratory tract infections within the first 6 years of life, was obtained through parental reports of diagnosed bronchitis or pneumonia (yes/no). The body mass index (BMI) at the 15‐year follow‐up was recorded by measured or reported weight and body size (kg/m^2^). Further confounding variables included allergic diseases (atopic eczema, allergic rhinitis) that have ever occurred up to the 15‐year follow‐up. Both are based on parent‐report regarding merged information of a medical diagnosis in the child's lifetime. Relevant dietary factors at age 15, such as total daily energy intake (kcal/day), as well as the proportion of starch, sucrose, fruits, and vegetables relative to total daily energy intake (% EI), were assessed using a self‐reported food frequency questionnaire.^31^

To investigate suspected puberty effects, particularly given previous studies emphasizing early puberty onset (≤10 years) as a risk factor for asthma,^32^, ^33^, ^34^ parent‐reported pubertal status at the 10‐year follow‐up was used. This determines whether the child already showed first signs of puberty, such as acne, pubic and armpit hair, breast development, and menstruation in girls, and penis and testicular enlargement in boys (yes/no).

### 2.5 Statistical analysis

Considering well‐known sex differences, both for asthma and psychopathological symptoms in youth,^6^, ^8^, ^9^ the sample is described separately for females and males. For descriptive analysis, counts and percentages for categorial variables and means (*M*) with standard deviations (SD) for continuous variables are presented. Sex differences were tested using Fisher's exact test for binary variables, chi‐squared test for categorial variables with more than two categories, and *t*‐test for continuous variables.

The prospective association of psychopathological symptoms (SDQ total score) as well as the association with each SDQ subscale separately at the age of 10 and current asthma (asthma/no asthma) at the age of 15 was analyzed by logistic regression. Regarding selected asthma endotypes (atopic/non‐atopic) and no asthma as reference, multinomial regression models were used. All models have been adjusted for the covariates and confounders listed above. In addition, an adjustment for asthma at 10 years and sensitivity analyses for new onset asthma after the 10‐year follow‐up were performed. Results are presented as odds ratio (OR) for logistic regression and relative risk ratio (RRR) for multinomial regression analyses with corresponding 95% confidence intervals (CI). To estimate effect modification by sex or pubertal status, interaction terms of the respective SDQ (sub‐)scale separately with sex or puberty onset were included in the adjusted models and followed by stratified analyses for these two factors if the interaction term was nominally significant. In addition, all regression models were examined separately for females and males. Sex‐specific analysis of the interaction between puberty onset and psychopathological symptoms on asthma in general and asthma endotypes was not feasible due to insufficient case numbers within each group.

For inferential statistics, a two‐sided significance level of .05 was assumed. All analyses were performed using IBM SPSS Statistics, version 28.^35^ Figures were created using the package Hmisc^36^ in R, version 4.1.3.^37^

## 3 RESULTS

**FIGURE 1:**
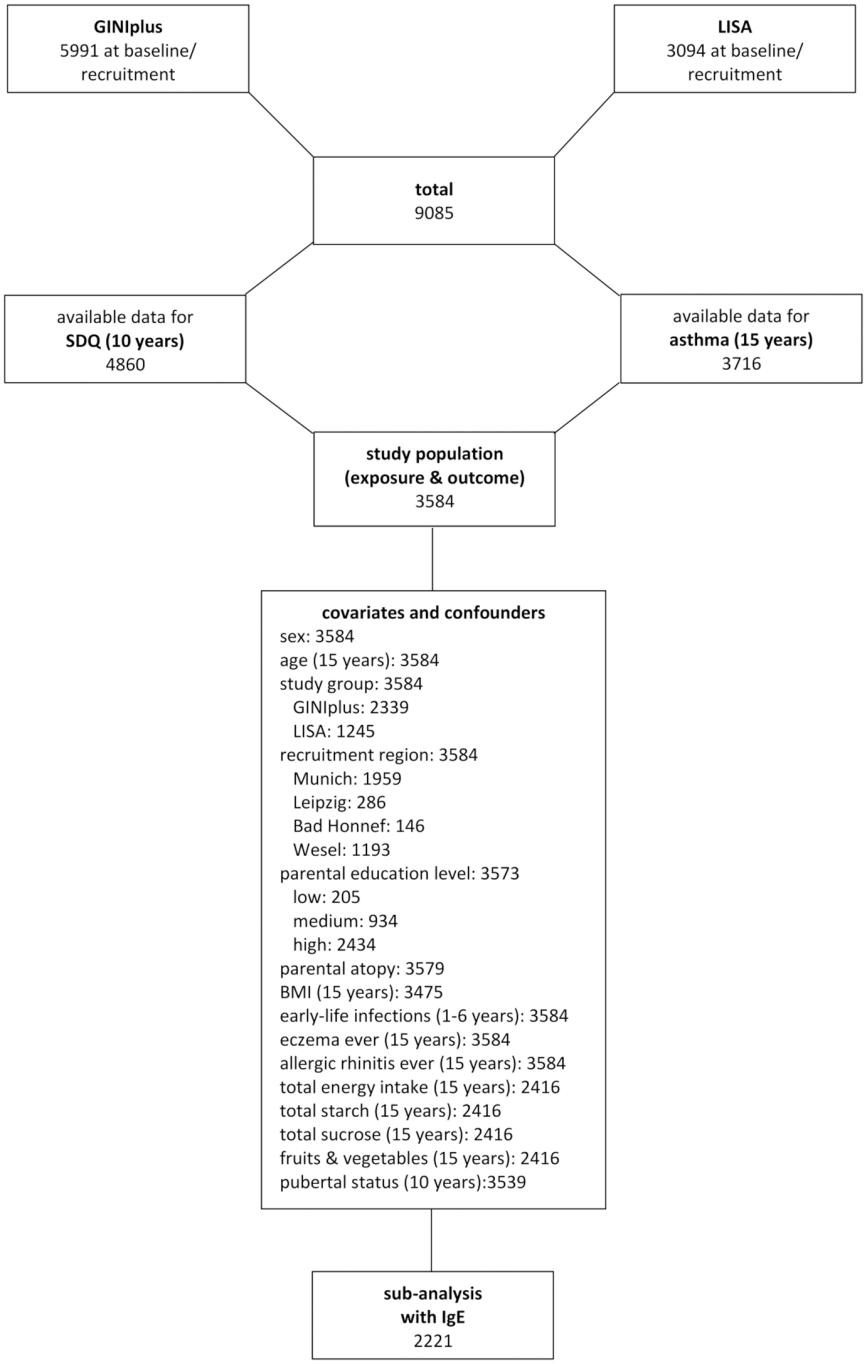
Derivation of analysis population.

Participants with available information on the SDQ total score at the 10‐year follow‐up and on current asthma at the 15‐year follow‐up were included. Differences between the original study population and the included population are detailed in Appendix S1. An overview of the sample characteristics is presented in Table 1. The study population comprises a total of 3584 participants (Figure 1). Of these, 203 (5.7%) suffered from current asthma at age 10 and 231 (6.4%) at age 15. Endotype‐specific asthma classification, including information on allergic sensitization, was implemented for 2185 participants in the 10‐year follow‐up and in 2221 participants in the 15‐year follow‐up (see online Appendices S2 and S3). At both measurement points, the majority of children and adolescents with current asthma and available IgE value was identified as atopic (10‐year follow‐up: 116, 77.3%; 15‐year follow‐up: 135, 81.8%), while non‐atopic asthma was comparatively less common (10‐year follow‐up: 34, 22.7%; 15‐year follow‐up: 30, 18.2%). Sex differences were evident, with more males being affected than females by current asthma in general at age 10 (*p* < .001) as well as at age 15 (*p* = .002). In contrast to the 10‐year follow‐up (*p* = .003), the 15‐year follow‐up did not reveal significant sex difference in the occurrence of atopic, non‐atopic asthma, and no asthma (*p* = .094). For the sensitivity analysis, 90 cases could be identified who did not report current asthma at age 10 years. Again, for new onset cases after age 10, no significant difference between the sexes could be detected (*p* = .749).

At the 10‐year follow‐up, psychopathological symptoms as assessed by the SDQ total score differed significantly between sexes, with a male predominance (males: 17.4%, females: 11.2%, *p* < .001). Furthermore, males showed significantly more hyperactivity and inattention, conduct problems, peer problems, as well as problems in prosocial behavior (each *p* < .001) than females. Sex differences were also evident regarding pubertal stage at age 10. Significantly more females (45.9%) exhibited early signs of puberty compared to males (11.4%), Table 1.

**TABLE 1:** Study population characteristics. Values presented as *n*/*N* (%) or mean (SD).

	Females (*n* = 1790)	Males (*n* = 1794)	*p*‐Value
*Baseline*			
Study group			
GINIplus	1187/1790 (66.3%)	1152/1794 (64.2%)	.194
Observation	718/1187 (60.5%)	670/1152 (58.2%)	
Intervention	469/1187 (39.5%)	482/1152 (41.8%)	
LISA	603/1790 (33.7%)	642/1793 (35.8%)	
Recruitment region			
Munich	971/1790 (54.2%)	988/1794 (55.1%)	.957
Leipzig	146/1790 (8.2%)	140/1794 (7.8%)	
Bad Honnef	74/1790 (4.1%)	72/1794 (4.0%)	
Wesel	599/1790 (33.5%)	594/1794 (33.1%)	
Parental education (proxy for SES)			
Low	95/1784 (5.3%)	110/1789 (6.1%)	.507
Medium	462/1784 (25.9%)	472/1789 (26.4%)	
High	1227/1784 (68.8%)	1207/1789 (67.5%)	
Parental atopy [yes vs. no]	1007/1787 (56.4%)	1022/1792 (57.0%)	.686
Early‐life infections [yes vs. no]	798/1790 (44.6%)	904/1794 (50.4%)	**<.001**

	10‐year follow‐up			15‐year follow‐up		
	Females (*n* = 1790)	Males (*n* = 1794)	*p*‐Value	Females (*n* = 1790)	Males (*n* = 1794)	*p*‐Value
*Follow‐ups*						
Onset of puberty [yes vs. no]	810/1765 (45.9%)	202/1774 (11.4%)	**<.001**			
SDQ total difficulties [borderline/abnormal vs. normal]	200/1790 (11.2%)	313/1794 (17.4%)	**<.001**			
Emotional problems	319/1790 (17.8%)	298/1794 (16.6%)	.353			
Conduct problems	158/1790 (8.8%)	248/1794 (13.8%)	**<.001**			
Hyperactivity/inattention	145/1790 (8.1%)	304/1794 (16.9%)	**<.001**			
Peer problems	112/1790 (6.3%)	166/1794 (9.3%)	**<.001**			
Problems in prosocial behavior	85/1790 (4.7%)	177/1794 (9.9%)	**<.001**			
Age				15.08 (.31)	15.07 (.30)	.239
BMI				20.15 (2.67)	20.39 (3.09)	.**016**
Eczema ever [yes vs. no]				517/1790 (28.9%)	512/1794 (28.5%)	.825
Allergic rhinitis ever [yes vs. no]				370/1790 (20.7%)	465/1794 (25.9%)	**<.001**
Total energy intake [kcal/day]				1779.28 (569.74)	2358.84 (669.73)	**<.001**
Total starch [% EI]				28.12 (7.26)	26.68 (7.49)	**<.001**
Total sucrose [% EI]				11.04 (4.12)	10.68 (4.11)	.**031**
Fruits and vegetables [% EI]				7.28 (5.16)	4.27 (3.03)	**<.001**
Asthma current^a^ [yes vs. no]	73/1768 (4.1%)	130/1768 (7.4%)	**<.001**	92/1790 (5.1%)	139/1794 (7.7%)	.**002**
Asthma endotype current^b^						
Atopic asthma	40/1086 (3.7%)	76/1099 (6.9%)	.**003**	56/1122 (5.0%)	79/1099 (7.2%)	.094
Non‐atopic asthma	17/1086 (1.6%)	17/1099 (1.5%)		16/1122 (1.4%)	14/1099 (1.3%)	
No asthma	1029/1086 (94.8%)	1006/1099 (91.5%)		1050/1122 (93.6%)	1006/1099 (91.5%)	

*Note*: *p*‐values were obtained from Fisher's exact test for binary variables, chi‐squared test for categorical variables with more than two categories and *t*‐test for continuous variables. Significant sex differences are highlighted in bold. Please note that the sample size from asthma current and asthma endotype differ because of specific inclusion criteria: ^a^positive parent‐reported medical diagnosis, whistling or wheezing symptoms, and medical treatment against asthma (at least two criteria); ^b^specific IgE levels.

Abbreviation: % EI, percentage of total daily energy intake.

**FIGURE 2:**
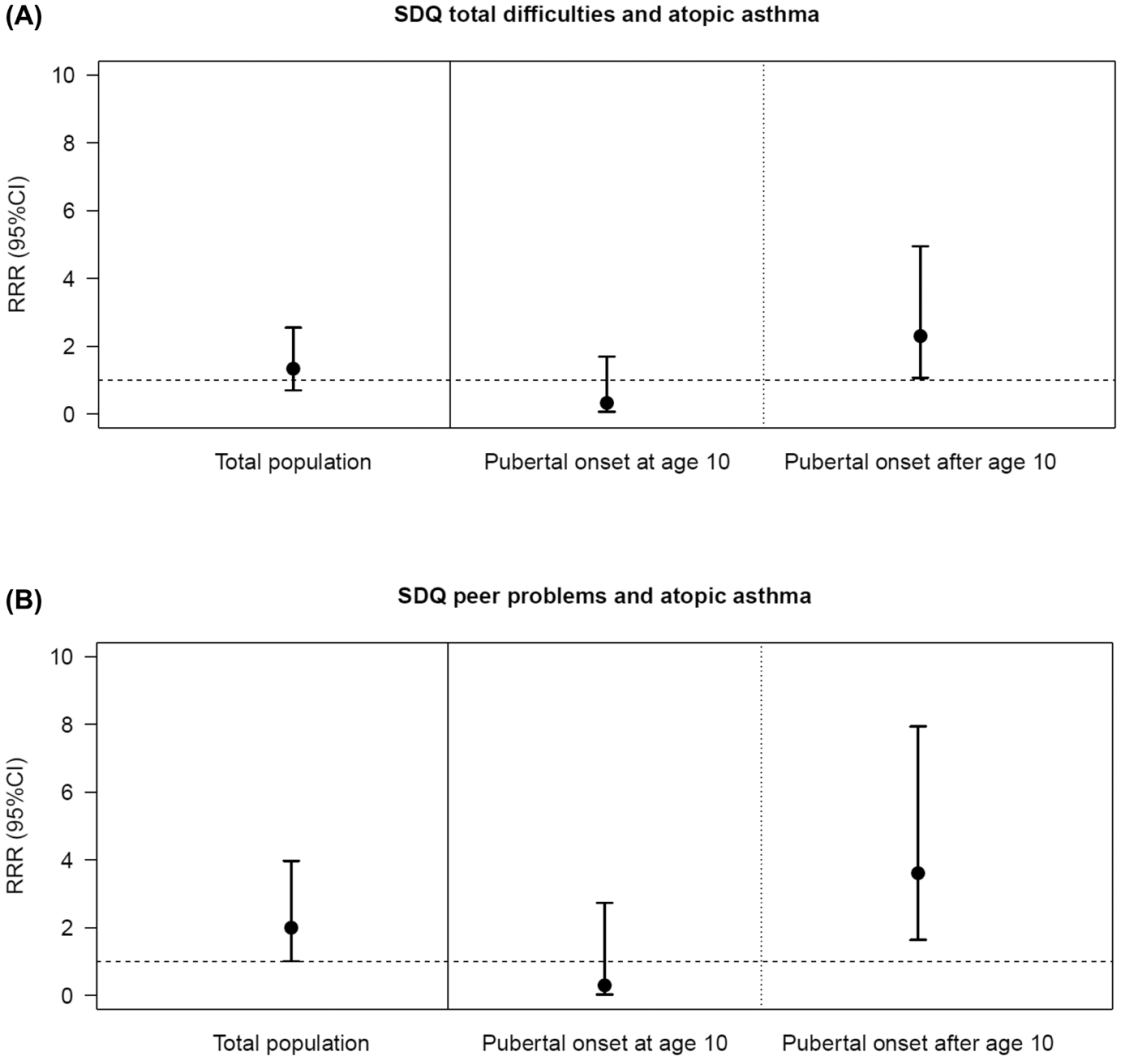
Significant Results of adjusted multinomial logistic regression analysis including the interaction term of SDQ (subscales), pubertal status at age 10 on atopic asthma at age 15 years.

The results on the prospective association (Table 2) showed an association of borderline/abnormal SDQ scores at age 10 with asthma at age 15 (OR = 1.69, 95% CI = 1.04–2.74, *p* = .035). When analyzing only asthma cases with onset after 10 years of age or adjusting for asthma status at age 10, the results did not reach significance; note, however, that the direction and magnitude of the effect were almost comparable (OR = 1.59, 95% CI = 0.77–3.29, *p* = .209 and OR = 1.54, 95% CI = 0.85–2.76, *p* = .152, respectively). Furthermore, in total, psychopathological symptoms in childhood were significantly associated with non‐atopic asthma in adolescence (RRR = 2.92, 95% CI = 1.13–7.60, *p* = .028), whereas there was no significant association with atopic asthma (*p* = .381). As shown in Table 2, there were no significant interactions for both models, neither with sex nor with puberty regarding the association between psychopathological symptoms in childhood and adolescent asthma in general. Similarly, regarding asthma endotypes, no significant interactions with sex were observed. However, there was a significant interaction of puberty onset with psychopathological symptoms in childhood on atopic asthma in adolescence (*p* = .020). Stratified analysis showed that psychopathological symptoms at age 10 significantly increased the likelihood of having atopic asthma at age 15 only in those with puberty onset after age 10 years, but not in those with onset of puberty at age 10 (Figure 2A).

**TABLE 2:** Adjusted^a^ logistic regression and multinomial regression models for the prospective association between SDQ at age 10 and asthma at age 15 compared to no asthma at age 15 (as reference).

	Current asthma 15 years (*n*/*N* = 231/3584)		Asthma endotypes 15 years			
			Atopic asthma (*n*/*N* = 135/2221)		Non‐atopic asthma (*n*/*N* = 30/2221)	
	OR^a^ (95% CI)	*p*‐Value	RRR^a^ (95% CI)	*p*‐Value	RRR^a^ (95% CI)	*p*‐Value
SDQ 10 years total difficulties^b^	**1.69 (1.04–2.74)**	.035	1.34 (0.70–2.55)*	.381	**2.92 (1.13–7.60)**	.028
New onset of asthma after 10 years (*n* = 90)						
SDQ 10 years total difficulties^b^	1.59 (0.77–3.29)	.209				
Adjusted for asthma 10 years						
SDQ 10 years total difficulties^b^	1.54 (0.85–2.76)	.152				

*Note*: Significant associations are highlighted in bold.

^a^ Covariates and confounders: sex, age (15 years), study group, recruitment region, parental education level, parental atopy, BMI, early‐life infections, eczema ever, allergic rhinitis ever, total energy intake (kcal/day), total starch (percentage of total daily energy intake, % EI), total sucrose (% EI), fruits & vegetables (% EI), pubertal status (10 years).

^b^ Borderline/abnormal versus normal.

* Significant interaction term of SDQ with onset of puberty (RRR = 0.13, 95% CI = 0.02–0.72, *p* = .020).

Regarding SDQ subscales (Table 3), peer problems at the age of 10 were associated with atopic asthma at age 15 (RRR = 2.00, 95% CI = 1.01–3.97, *p* = .048). For adolescent asthma in general (*p* = .083) and non‐atopic asthma (*p* = .456), no significant relationship was evident. Additionally, a significant interaction effect on atopic asthma was found for peer problems and puberty (*p* = .029), indicating that children who suffered from peer problems and who had not yet started puberty at age 10 were more likely to have atopic asthma in adolescence (Figure 2B). Again, no significant interactions with sex were found for asthma in general or asthma endotypes.

**TABLE 3:** Adjusted^a^ logistic regression and multinomial regression models for the prospective associations between each SDQ subscale at age 10 and asthma at age 15 compared to no asthma at age 15 (as reference).

	Current asthma 15 years (*n*/*N* = 231/3584)		Asthma endotypes 15 years			
			Atopic asthma (*n*/*N* = 135/2221)		Non‐atopic asthma (*n*/*N* = 30/2221)	
	OR^a^ (95% CI)	*p*‐Value	RRR^a^ (95% CI)	*p*‐Value	RRR^a^ (95% CI)	*p*‐Value
SDQ 10 years emotional problems^b^	1.50 (0.93–2.43)	.099	1.68 (0.90–3.17)	.106	1.83 (0.68–4.90)	.230
SDQ 10 years conduct problems^b^	0.87 (0.47–1.61)	.664	0.70 (0.31–1.57)	.386	0.73 (0.16–3.34)	.689
SDQ 10 years hyperactivity/inattention^b^	1.20 (0.68–2.11)	.537	0.63 (0.27–1.44)	.269	2.38 (0.79–7.16)	.123
SDQ 10 years peer problems^b^	1.66 (0.94–2.92)	.083	**2.00 (1.01–3.97)** *	.048	1.63 (0.45–5.84)	.456
SDQ 10 years problems in prosocial behavior^b^	1.12 (0.56–2.24)	.741	141 (0.58–3.44)	.455	0.59 (0.08–4.66)	.618

*Note*: Significant associations are highlighted in bold.

^a^ Covariates and confounders: sex, age (15 years), study group, recruitment region, parental education level, parental atopy, BMI, early‐life infections, eczema ever, allergic rhinitis ever, total energy intake (kcal/day), total starch (percentage of total daily energy intake, % EI), total sucrose (% EI), fruits & vegetables (% EI), pubertal status (10 years).

^b^ Borderline/abnormal versus normal.

* Significant interaction term of SDQ with onset of puberty (RRR = 0.08, 95% CI = 0.01–0.78, *p* = .029).

Sex‐specific results regarding the prospective association between psychopathological symptoms in childhood and asthma in adolescence are presented in Appendix S5.

## 4 DISCUSSION

### 4.1 Summary of main findings

Based on a large‐scale prospective investigation, we found an association of psychopathological symptoms in childhood with asthma in adolescence. This prospective association was specifically detected for non‐atopic asthma. Considering the SDQ subscales, peer problems in childhood were related to atopic asthma in adolescence. The prevalence of atopic asthma in adolescence was increased in those with psychopathological symptoms in childhood, specifically with peer problems, and with puberty onset after age 10.

### 4.2 Prospective association between psychopathological symptoms and asthma

The results of the prospective analyses investigating the relationship between psychopathological symptoms at age 10 and asthma at age 15 are in line with our hypotheses and correspond to the findings of previous longitudinal studies in adults.^14^, ^15^, ^16^ One explanation for the association between psychopathological symptoms and asthma assumes that mental health problems are often accompanied by a limited quality of life.^38^ This can contribute to increased difficulties in managing important developmental milestones during youth, such as gaining autonomy and exploring identity.^39^ Given that peer interactions play an important role in identity formation and social integration, they may contribute to the association between peer problems and atopic asthma, as peer problems can result in chronic stress, which in turn can lead to a disturbed regulation of the hypothalamic–pituitary–adrenal (HPA) and sympathetic‐adrenal‐medullary (SAM) axes and altered regulation of inflammatory response and lung function,^40^, ^41^ potentially promoting the persistence or exacerbation of atopic asthma.

While short‐term release of stress hormones such as cortisol, epinephrine, and norepinephrine has an anti‐inflammatory effect, long‐term stress, as, for example, caused by psychopathological problems, leads to an overstimulation of neuronal and hormonal pathways, and corresponding receptors (e.g., glucocorticoid receptors) in the immune system are down‐regulated.^11^, ^42^, ^43^ In addition, stress has a stimulating effect on the production of certain cytokines (e.g., interleukin (IL)‐4, IL‐5, IL‐13) associated with asthma.^11^, ^43^, ^44^ This leads to a reduced regulation of inflammatory reactions to asthma triggers such as respiratory infections, known as an important risk factor for non‐atopic asthma.^41^, ^42^ While atopic asthma develops by allergic sensitization,^12^ non‐atopic asthma is often triggered by environmental risk factors such as (environmental) tobacco‐smoke exposure.^45^, ^46^ Given the close link between (second‐hand) smoking and low SES, it poses a risk in itself.^45^ Also, in the development and maintenance of psychopathological symptoms, a low SES is considered a risk factor,^46^, ^47^ so residual confounding cannot be ruled out.

It should be discussed that the relationship between psychopathological symptoms and asthma did not remain significant when only new asthma cases in adolescence were considered or when the analyses were adjusted for asthma during childhood. However, it needs to be highlighted that this finding is related to the rather low number of new asthma cases (*n* = 90) and thus the restricted statistical power of these additional analyses, as the magnitude and direction of effects remained stable.

### 4.3 Interaction effects of sex and puberty

We found that children at age 10 with psychopathological symptoms, especially peer problems, who have not entered puberty are more likely to suffer from atopic asthma in adolescence. These findings support previous research, with studies placing special focus on sex hormones as influencing factors for asthma.^8^, ^32^, ^48^, ^49^ There is evidence that female sex hormones are particularly relevant.^50^, ^51^ In this respect, menarche is discussed as a risk factor for asthma.^33^, ^49^, ^51^ It must be mentioned that early menarche, typically defined as below the age of 11 years,^49^ occurs in less than 10% of girls,^52^ even if first signs of puberty onset are already visible. Therefore, the influence of female sex hormones at the age of 10 plays a less important role. Even though no significant interaction with sex was observed, the results of the sex‐specific analyses (see Appendix S5), indicating specific significant associations between psychopathological symptoms and asthma in general and asthma endotypes in males but not in females, may offer a possible explanation through the presumed protective effect of the male sex hormone testosterone.^48^ In case of pending onset of puberty at 10 years, this impact may not have yet begun. Puberty development begins later in boys than girls, and the majority of boys enter puberty after the age of 10.^53^ Coupled with peer problems, which make it difficult to integrate into a social group and develop identity appropriately, boys at age 10 might show an increased vulnerability to stress and thus to the development, persistence, or exacerbation of atopic asthma.

However, it should be considered in all interpretations that the CIs are partly large and overlapping, which may potentially affect the statistical significance. As the influence of psychopathological symptoms and pre‐pubertal mechanisms on asthma endotypes has received little attention in childhood and adolescence, future studies are needed to support our explanations.

### 4.4 Strengths and limitations

The main strength of the study is the large‐scale, population‐based sample, which enhances the potential for greater generalizability of the findings. Moreover, the prospective design addresses previous research gaps with respect to the directionality of the association between psychopathological symptoms and asthma in childhood and adolescence, although this does not allow for fully establishing causality. The consideration of atopy status is also an important strength of the study as there are well‐known differences in the etiology and the disease courses in distinct asthma endotypes, but little is known about differential influences of psychosocial factors therein. Additionally, examining various psychopathological symptom domains like in the present study provides a comprehensive picture and allows the identification of specific psychopathological risk factors in childhood for the occurrence of asthma in adolescents, which in turn enables important starting points for targeted prevention approaches. The thorough control of potential confounders is also a positive aspect, particularly since few previous studies apply such a detailed adjustment.

Despite these strengths, there are also some limitations of the study, including the fact that all (birth) cohort studies are subject to natural dynamics and suffer from loss to follow‐up,^54^ which may contribute to selection bias. As in any longitudinal cohort study, most participants, in our study population the parents, have a high level of education, which serves as a proxy for SES.^55^ Given that a low SES increases both the risk of psychopathological symptoms^56^ and asthma,^5^ the strength of the associations found may be underestimated. As this is a population‐based study whose strengths have already been listed above, another critical aspect concerns the relatively small size of the asthma group, both for asthma in general and selected asthma endotypes, compared to the no asthma reference group, which may compromise the stability of the estimates and potentially obscure actual effects. Nevertheless, the asthma distribution in our study is consistent with data reported from other German cohort studies on child and adolescent health (e.g., KiGGS wave 2^38^). Another criticism might arise due to the use of parent‐reports for the assessment of asthma, where some degree of misclassification due to the lack of medical expertise cannot be ruled out. However, sensitivity analyses, incorporating objective data from FeNO measurement and a positive spirometric bronchodilation response, provide substantial support for our results. The use of parent‐reports in assessing pubertal status at age 10 might in some cases lead to misclassification. It is important to note, however, that the use of parental reports to capture early pubertal development in large epidemiological studies is recommended, as questions pertinent to young children may be too difficult to answer, and conducting a physical examination is typically not feasible within these large‐scale studies.^57^ A similar limitation might arise from the SDQ completed by parents, who might underestimate internalizing symptoms and overestimate externalizing symptoms.^58^ In this context, it should be considered that studies show moderate to strong correlations between self‐report and parent‐report^59^, ^60^ and that the distribution of the SDQ largely aligns with pre‐pandemic data from other German and European studies,^61^, ^62^ as the 10‐year and 15‐year follow‐ups of our study were also conducted before COVID‐19.

## 5 CONCLUSION AND FUTURE PERSPECTIVES

Our study provides important new insights regarding the prospective relationship between psychopathological symptoms and asthma in childhood and adolescence. The findings imply that different forms of asthma should receive a greater consideration in these age groups. In practice, our results suggest that the occurrence of non‐atopic asthma may be mitigated through the early identification of psychopathological symptoms and suitable interventions. While the avoidance of allergen exposure has been established as a preventive and treatment strategy for the occurrence of atopic asthma, the specific association with peer problems also underscores the need for interventions aimed at coping with social difficulties to minimize the risk of persistence and exacerbation of this asthma endotype. To confirm the robustness of our results, future studies should include an even larger sample size and, ideally, rely on the same informant regarding psychopathological symptoms across the follow‐ups (e.g., include only self‐reports), which would also enable the use of trajectory‐based models for more nuanced insights into the associations. Additionally, to gain a better understanding of the complex interaction between psychopathological symptoms and asthma endotypes, future research should examine self‐perceived stress as a possible linking factor.

Our study additionally provides evidence that both sex and particularly puberty play an important role in children with psychopathological symptoms and the risk of atopic asthma in adolescence. This leads to a new research gap because little is known about pre‐pubertal hormonal mechanisms of action, particularly for asthma endotypes. Future studies should include specific IgE levels, sex‐specific bio‐psycho‐social developmental status, and hormones to achieve greater insights.

## AUTHOR CONTRIBUTIONS

**Pia‐Marie Keim:** Conceptualization; methodology; formal analysis; writing – original draft; writing – review and editing; visualization. **Ellen Greimel:** Conceptualization; formal analysis; supervision; writing – original draft; methodology; project administration; writing – review and editing. **Lisa Feldmann:** Conceptualization; formal analysis; writing – review and editing. **Charlotte Elisabeth Piechaczek:** Conceptualization; formal analysis; writing – review and editing. **Carla P. Harris:** Writing – review and editing. **Claudia Flexeder:** Writing – review and editing. **Dietrich Berdel:** Resources; funding acquisition; investigation; writing – review and editing. **Andrea von Berg:** Resources; funding acquisition; investigation; writing – review and editing. **Sibylle Koletzko:** Resources; funding acquisition; investigation; writing – review and editing. **Carl‐Peter Bauer:** Resources; funding acquisition; investigation; writing – review and editing. **Tamara Schikowski:** Data curation; resources; funding acquisition; investigation; writing – review and editing. **Gunda Herberth:** Resources; funding acquisition; investigation; writing – review and editing. **Joachim Heinrich:** Data curation; resources; funding acquisition; investigation; writing – review and editing. **Gerd Schulte‐Körne:** Conceptualization; methodology; project administration; writing – review and editing. **Marie Standl:** Conceptualization; data curation; resources; formal analysis; supervision; writing – original draft; methodology; writing – review and editing; project administration.

## FUNDING INFORMATION

GINIplus was mainly covered by the Federal Ministry for Education, Science, Research and Technology (grant number 01EE9401‐4) (intervention arm) and Helmholtz Zentrum Munich (former GSF) (observation arm) for the first 3 years. The 4‐year, 6‐year, 10‐year, and 15‐year follow‐up examinations were funded by the respective budgets of the 5 study centres (Helmholtz Zentrum Munich (former GSF), Research Institute at Marien‐Hospital Wesel, LMU Munich, TU Munich, and from 6 years onwards also from IUF—Leibniz Research‐Institute for Environmental Medicine at the University of Düsseldorf and grants by the Federal Ministry for Environment (IUF Düsseldorf, grant number 20462296)). The 15‐year follow‐up examination was additionally supported by the Commission of the European Communities, the 7th Framework Program: MeDALL project (grant number 261357), and by the companies Mead Johnson and Nestlé. LISA was mainly covered from the Federal Ministry for Education, Science, Research and Technology (grant number 01EG9705/2 and 01EG9732) and Helmholtz Zentrum Munich (former GSF), Helmholtz‐Centre for Environmental Research—UFZ, Leipzig, Research Institute at Marien‐Hospital Wesel, Pediatric Practice, Bad Honnef for the first 2 years. The 4‐year, 6‐year, 10‐year and 15‐year follow‐up examinations were funded by the respective budgets of the involved partners (Helmholtz Zentrum Munich (former GSF), Helmholtz‐Centre for Environmental Research—UFZ, Leipzig, Research Institute at Marien‐Hospital Wesel, Pediatric Practice, Bad Honnef, IUF – Leibniz‐Research Institute for Environmental Medicine at the University of Düsseldorf) and in addition by a grant from the Federal Ministry for Environment (IUF Düsseldorf, grant number 20462296). The 15‐year follow‐up examination was additionally supported of the Commission of the European Communities, the 7th Framework Program: MeDALL project (grant number 261357). MS received funding from the European Research Council (ERC) under the European Union's Horizon 2020 research and innovation programme (grant agreement No. 949906).

## CONFLICT OF INTEREST STATEMENT

The authors declare none.

## PEER REVIEW

The peer review history for this article is available at https://www.webofscience.com/api/gateway/wos/peer‐review/10.1111/pai.70151.

## ETHICS STATEMENT

The GINIplus and the LISA Study were approved by the local ethics committees (Bavarian Board of Physicians, Board of Physicians of North‐Rhine‐Westphalia, Board of Physicians of Saxony). The authors assert that all procedures contributing to this work comply with the ethical standards of the relevant national and institutional committees on human experimentation and with the Helsinki Declaration of 1975, as revised in 2008.

## Supporting information


Appendix S1.

Appendix S2.

Appendix S3.

Appendix S4.

Appendix S5.

Appendix S6.

## Data Availability

Due to data protection reasons, the datasets analyzed during the current study cannot be made publicly available. The datasets are available to interested researchers from Marie Standl (Email: marie.standl@helmholtz-munich.de) on reasonable request (e.g., reproducibility), provided the release is consistent with the consent given by the GINIplus and LISA study participants. Ethical approval might be obtained for the release, and a data transfer agreement from the legal department of the Helmholtz Zentrum Munich must be accepted.
