# Supplementary material for: Prospective association between psychopathological symptoms in childhood and asthma in adolescence: Results from the GINIplus and LISA birth cohort studies
Source: Pediatr Allergy Immunol. 2025 Jul 24;36(7):e70151. doi: 10.1111/pai.70151 (PMC12287888; doi:10.1111/pai.70151)
Supplement: Supplementary file 1 — Appendix S1. [file PAI-36-e70151-s005.docx]

## Supplement S1. Comparison of the analysis population and total population.

Table S1. Values presented as n/N (%) or mean (SD). Significant differences within total population and included population are highlighted in bold.

| ***baseline*** |  | | |  | | | |  | |  |
| --- | --- | --- | --- | --- | --- | --- | --- | --- | --- | --- |
|  | **total population**  *N*=9085 | | | **included in analysis**  (exposure & outcome)  *n*=3584 | | | | *p*-value | |  |
| **sex** |  | | |  | | | |  | |  |
| females | 4349/8924 (48.7%) | | | 1790/3584 (49.9%) | | | | .228 | |  |
| males | 4575/8924 (51.3%) | | | 1794/3584 (50.1%) | | | |  | |  |
| **study group** |  | | |  | | | | **.027** | |  |
| GINIplus | 5991/9085 (65.9%) | | | 2339/3584 (65.3%) | | | |  | |  |
| observation | 3739/5991 (62.4%) | | | 1388/2339 (59.3%) | | | |  | |  |
| intervention | 2252/5991 (37.6%) | | | 951/2339 (40.7%) | | | |  | |  |
| LISA | 3094/9085 (34.1%) | | | 1245/3584 (34.7%) | | | |  | |  |
| **recruitment region** |  | | |  | | | | **<.001** | |  |
| Munich | 4413/9085 (48.6%) | | | 1959/3584 (54.7%) | | | |  | |  |
| Leipzig | 976/9085 (10.7%) | | | 286/3584 (8.0%) | | | |  | |  |
| Bad Honnef | 306/9085 (3.4%) | | | 146/3584 (4.1%) | | | |  | |  |
| Wesel | 3390/9085 (37.3%) | | | 1193/3584 (33.3%) | | | |  | |  |
| **parental education** (proxy for SES) |  | | |  | | | | **<.001** | |  |
| low | 969/9004 (10.8%) | | | 205/3573 (5.7%) | | | |  | |  |
| medium | 2656/9004 (29.5%) | | | 934/3573 (26.1%) | | | |  | |  |
| high | 5379/9004 (59.7%) | | | 2434/3573 (68.1%) | | | |  | |  |
| **parental atopy**  [yes vs. no] | 4841/9069 (53.4%) | | | 2029/3579 (56.7%) | | | | **<.001** | | |
| **early-life infections**  [yes vs. no] | 3511/7700 (45.6%) | | | 1702/3584 (47-5%) | | | | .062 | | |
| ***follow-ups*** | | | | | | | | | |  |
|  | 10-year follow-up | | | | 15-year follow-up | | | | |  |
|  | **total population**  *N*=9085 | **in analysis**  (exposure & outcome)  *n*=3584 | *p*-value | | | **total population**  *N*=9085 | **in analysis**  (exposure & outcome)  *n*=3584 | | *p*-value | |
| **onset of puberty**  [yes vs. no] | 1484/4968  (29.9%) | 1012/3539  (28.6%) | .209 | | |  |  | |  | |
| **SDQ**  **total difficulties**  [borderline/ abnormal vs. normal] | 738/4860  (15.2%) | 513/3584  (14.3%) | .278 | | |  |  | |  | |
| emotional problems | 871/4861  (17.9%) | 617/3584  (17.2%) | .419 | | |  |  | |  | |
| conduct problems | 574/4861  (11.8%) | 406/3584  (11.3%) | .514 | | |  |  | |  | |
| hyperactivity/ inattention | 654/4861  (13.5%) | 449/3584  (12.5%) | .215 | | |  |  | |  | |
| peer problems | 423/4860  (8.7%) | 278/3584  (7.8%) | .120 | | |  |  | |  | |
| problems in prosocial behavior | 366/4862  (7.5%) | 262/3584  (7.3%) | .737 | | |  |  | |  | |
| **age** |  |  |  | | | 15.10  (.32) | 15.07  (.30) | | **<.001** | |
| **BMI** |  |  |  | | | 20.38  (2.97) | 20.27  (2.89) | | .073 | |
| **eczema ever**  [yes vs. no] |  |  |  | | | 1950/7704  (25.3%) | 1029/3584  (28.7%) | | **<.001** | |
| **allergic rhinitis ever**  [yes vs. no] |  |  |  | | | 1301/6741  (19.3%) | 835/3584  (23.3%) | | **<.001** | |
| **total energy intake** [kcal/day] |  |  |  | | | 2053.1  (693.82) | 2050.83  (682.78) | | .902 | |
| **total starch** [%EI*] |  |  |  | | | 27.41  (7.45) | 27.44  (7.40) | | .865 | |
| **total sucrose** [%EI*] |  |  |  | | | 10.86  (4.10) | 10.87  (4.12) | | .888 | |
| **fruits & vegetables** [%EI*] |  |  |  | | | 5.86  (4.72) | 5.87  (4.55) | | .926 | |
| **asthma current**^a^  [yes vs. no] | 264/4954  (5.3%) | 203/3536  (5.7%) | .412 | | | 258/3716  (6.9%) | 231/3584  (6.4%) | | .400 | |
| **asthma endotype current**^b^ |  |  | .877 | | |  |  | | .793 | |
| atopic asthma | 142/2844  (5.0%) | 116/2185  (5.3%) |  | | | 150/2293  (6.5%) | 135/2221  (6.1%) | |  | |
| non-atopic asthma | 44/2844  (1.5%) | 34/2185  (1.6%) |  | | | 33/2293  (1.4%) | 30/2221  (1.4%) | |  | |
| no asthma | 3658/2884  (93.5) | 2035/2185  (93.1%) |  | | | 2110/2293  (92.0%) | 2056/2221  92.6%) | |  | |

*p*-values were obtained from Fisher´s exact test for binary variables, chi-squared test for categorical variables with more than two categories and t-test for continuous variables.

Please note that the sample size from asthma current and asthma endotype differ because of specific inclusion criteria: ^a^positive parent-reported medical diagnosis, whistling or wheezing symptoms and medical treatment against asthma (at least two criteria); ^b^specific IgE levels; *%EI: percentage of total daily energy intake.

As shown in Table S1, the population included in the present analysis (available information on the SDQ total score at 10-year follow-up and on current asthma in the 15-year follow-up) differed from the original study population in study characteristics (study group: *p*<.027; recruitment region: *p*<.001), particularly due to a higher proportion of participants in the GINIplus intervention group and from Munich and Bad Honnef in the analysis sample. The observed difference in parental education level (proxy for SES) (*p*<.001) could be attributed to participants from Munich generally exhibiting a higher educational level than those from other recruitment regions (Munich: 59.9%, Leipzig: 8.7%, Bad Honnef: 3.7%, Wesel: 27.7%; *p*<.001). Furthermore, the 15-year follow-up revealed significant differences in ever being affected by allergic rhinitis (*p*<.001) or atopic eczema (*p*<.001). One possible explanation is that both diseases are closely linked to asthma, and therefore also to our inclusion criteria. Finally, at this point it should be emphasized had the included sample does not differ in relevant terms of the SDQ (subscales) as the independent variable of interest or asthma in general and asthma endotypes as dependent variables, reinforcing the robustness of our findings.
