# Supplementary material for: Prospective association between psychopathological symptoms in childhood and asthma in adolescence: Results from the GINIplus and LISA birth cohort studies
Source: Pediatr Allergy Immunol. 2025 Jul 24;36(7):e70151. doi: 10.1111/pai.70151 (PMC12287888; doi:10.1111/pai.70151)
Supplement: Supplementary file 2 — Appendix S2. [file PAI-36-e70151-s006.docx]

## Supplement S2. Characteristics of study population included for sub-analyses vs. excluded study population.

Table S2. Values presented as n/N (%) or mean (SD). Significant differences within included and excluded subjects for sub-analysis are highlighted in bold.

|  |  | **sub-population**  **(with IgE)** ^a^  *n*=2221 | **study population**  **without IgE**^b^  *n*=1363 | *p*-value^¶^ |
| --- | --- | --- | --- | --- |
| ***baseline*** |  |  |  |  |
| **sex** |  |  |  | .390 |
| females |  | 1122/2221 (50.5%) | 668/1363 (49.0%) |  |
| males |  | 1099/2221 (49.5%) | 695/1363 (51.0%) |  |
| **study group** |  |  |  | .386 |
| GINIplus |  | 1437/2221 (64.7%) | 902/1363 (66.2%) |  |
| observation |  | 754/1437 (52.5%) | 634/902 (70.3%) |  |
| intervention |  | 683/1437 (47.5%) | 268/902 (29.7%) |  |
| LISA |  | 784/2221 (35.3%) | 461/1363 (33.8%) |  |
| **recruitment region** |  |  |  | **<.001** |
| Munich |  | 1232/2221 (55.5%) | 727/1363 (53.3%) |  |
| Leipzig |  | 204/2221 (9.2%) | 82/1363 (6.0%) |  |
| Bad Honnef |  | 95/2221 (4.3%) | 51/1363 (3.7%) |  |
| Wesel |  | 690/2221 (31.1%) | 503/1363 (36.9%) |  |
| **parental education** (proxy for SES) |  |  |  | **<.001** |
| low |  | 107/2214 (4.8%) | 98/1359 (7.2%) |  |

| medium |  | 543/2214 (24.5%) | 391/1359 (28.8%) |  |
| --- | --- | --- | --- | --- |
| high |  | 1564/2214 (70.6%) | 870/1359 (64.0%) |  |
| **parental atopy**  [yes vs. no] |  | 1319/2219 (59.4%) | 710/1360 (52.2%) | **<.001** |
| **early-life infections**  [yes vs. no] |  | 1097/2221 (49.4%) | 605/1363 (44.4%) | **.004** |
| ***10-year follow-up*** |  |  |  |  |
| **onset of puberty**  [yes vs. no] |  | 625/2221 (28.5%) | 387/1344 (28.8%) | .848 |
| **SDQ**  **total difficulties**  [borderline/ abnormal vs. normal] |  | 332/2221 (14.9%) | 181/1363 (13.3%) | .169 |
| emotional problems |  | 383/2221 (17.2%) | 234/1363 (17.2%) | .964 |
| conduct problems |  | 266/2221 (12.0%) | 140/1363 (10.3%) | .128 |
| hyperactivity/ inattention |  | 286/2221 (12.9%) | 163/1363 (12.0%) | .436 |
| peer problems |  | 187/2221 (8.4%) | 91/1363 (6.7%) | .062 |
| problems in prosocial behavior |  | 149/2221 (6.7%) | 113/1363 (8.3%) | .086 |
| ***15-year follow-up*** |  |  |  |  |
| **age** |  | 15.03 (.26) | 15.14 (.35) | **<.001** |
| **BMI** |  | 20.24 (2.84) | 20.32 (2.97) | .421 |
| **eczema ever**  [yes vs. no] |  | 677/2221 (30.5%) | 352/1363 (25.8%) | **.003** |
| **allergic rhinitis ever**  [yes vs. no] |  | 566/2221 (25.5%) | 269/1363 (19.7%) | **<.001** |
| **total energy intake** [kcal/day] |  | 2065.83 (678.43) | 2015.34 (692.12) | .097 |
| **total starch** [%EI*] |  | 27.51 (7.34) | 27.28 (7.55) | .485 |
| **total sucrose** [%EI*] |  | 10.78 (4.04) | 11.10 (4.28) | .081 |
| **fruits & vegetables**[%EI*] |  | 5.82 (4.53) | 5.99 (4.58) | .417 |

*p*-values were obtained from Fisher´s exact test for binary variables, chi-squared test for categorical variables with more than two categories and t-test for continuous variables.

^a^ available data for SDQ at age 10 and asthma-endotype (based on IgE) at age 15.

^b^ included for main analysis but excluded for sub-analysis.

* %EI: percentage of total daily energy intake.

Differences between included and excluded subjects for the sub-analysis (endotype-specific asthma group) could result, among other things, from the fact that on-site examinations, as is necessary for IgE determination, are more attended by subjects with a higher socioeconomic status (measured by parental education) ^55^ (*p*<.001). Here you can find a link to the recruitment region (*p*<.001). In the sub-population there are fewer participants from Wesel, where the proportion of subjects with a high level of parental education is comparatively lower. In addition, increased concern resulting from the participants´ medical history (early-life infections affecting the lower respiratory tract, *p*=.004), own allergic diseases as eczema ever (*p*=.003) and allergic rhinitis ever (*p*<.001), or those of their parents (parental atopy, *p*<.001) could have led to a higher willingness to participate. The significant difference in age (*p*<.001) is so marginal that it is negligible (95%CI=-.14, -.10).
