# Supplementary material for: Prospective association between psychopathological symptoms in childhood and asthma in adolescence: Results from the GINIplus and LISA birth cohort studies
Source: Pediatr Allergy Immunol. 2025 Jul 24;36(7):e70151. doi: 10.1111/pai.70151 (PMC12287888; doi:10.1111/pai.70151)
Supplement: Supplementary file 3 — Appendix S3. [file PAI-36-e70151-s002.docx]

## Supplement S3. Study sub-population characteristics for endotype-specific asthma-group.

Table S3. Values presented as n/N (%) or mean (SD). Significant sex differences are highlighted in bold.

| ***baseline*** |  | | |  | | | |  | |  |
| --- | --- | --- | --- | --- | --- | --- | --- | --- | --- | --- |
|  | **females** (*n*=1122) | | | **males** (*n*=1099) | | | | *p*-value | |  |
| **study group** |  | | |  | | | | .156 | |  |
| GINIplus | 742/1122 (66.1%) | | | 695/1099 (63.2%) | | | |  | |  |
| observation | 718/1187 (60.5%) | | | 670/1152 (58.2%) | | | |  | |  |
| intervention | 469/1187 (39.5%) | | | 482/1152 (41.8%) | | | |  | |  |
| LISA | 380/1122 (33.9%) | | | 404/1099 (36.8%) | | | |  | |  |
| **recruitment region** |  | | |  | | | | .744 | |  |
| Munich | 616/1122 (54.9%) | | | 616/1099 (56.1%) | | | |  | |  |
| Leipzig | 100/1122 (8.9%) | | | 104/1099 (9.5%) | | | |  | |  |
| Bad Honnef | 46/1122 (4.1%) | | | 49/1099 (4.5%) | | | |  | |  |
| Wesel | 360/1122 (32.1%) | | | 330/1099 (30.0%) | | | |  | |  |
| **parental education** (proxy for SES) |  | | |  | | | | .981 | |  |
| low | 53/1118 (4.7%) | | | 54/1096 (4.9%) | | | |  | |  |
| medium | 275/1118 (24.6%) | | | 268/1096 (24.5%) | | | |  | |  |
| high | 790/1118 (70.7%) | | | 774/1096 (70.6%) | | | |  | |  |
| **parental atopy**  [yes vs. no] | 667/1121 (59.5%) | | | 652/1098 (59.4%) | | | | .966 | | |
| **early-life infections**  [yes vs. no] | 518/1122 (46.2%) | | | 579/1009 (52.7%) | | | | **.002** | | |
| ***follow-ups*** | | | | | | | | | |  |
|  | 10-year follow-up | | | | 15-year follow-up | | | | |  |
|  | **females**  (*n*=1122) | **males**  (*n*=1099) | *p*-value | | | **females**  (*n*=1122) | **males**  (*n*=1099) | | *p*-value | |
| **onset of puberty**  [yes vs. no] | 516/1105  (46.7%) | 109/1099  (10.0%) | **<.001** | | |  |  | |  | |
| **SDQ**  **total difficulties**  [borderline/ abnormal vs. normal] | 124/1122  (11.1%) | 208/1099  (18.9%) | **<.001** | | |  |  | |  | |
| emotional problems | 204/1122  (18.2%) | 179/1099  (16.3%) | .239 | | |  |  | |  | |
| conduct problems | 105/1122  (9.4%) | 161/1099  (14.6%) | **<.001** | | |  |  | |  | |
| hyperactivity/ inattention | 87/1122  (7.8%) | 199/1099  (18.1%) | **<.001** | | |  |  | |  | |
| peer problems | 79/1122  (7.0%) | 108/1099  (9.8%) | **.022** | | |  |  | |  | |
| problems in prosocial behavior | 50/1122  (4.5%) | 99/1099  (9.0%) | **<.001** | | |  |  | |  | |
| **age** |  |  |  | | | 15.03  (.27) | 15.02  (.25) | | .528 | |
| **BMI** |  |  |  | | | 20.19  (2.64) | 20.29  (3.03) | | .394 | |
| **eczema ever**  [yes vs. no] |  |  |  | | | 346/1122  (30.8%) | 331/1099  (30.1%) | | .747 | |
| **allergic rhinitis ever**  [yes vs. no] |  |  |  | | | 258/1122  (23.0%) | 308/1099  (28.0%) | | **.007** | |
| **total energy intake** [kcal/day] |  |  |  | | | 1789.49  (558.50) | 2376.76  (667.06) | | **<.001** | |
| **total starch** [%EI*] |  |  |  | | | 28.17  (7.19) | 26.77  (7.45) | | **<.001** | |
| **total sucrose** [%EI*] |  |  |  | | | 10.95  (3.98) | 10.58  (4.11) | | .063 | |
| **Fruits & vegetables** [%EI*] |  |  |  | | | 7.15  (5.17) | 4.33  (3.08) | | **<.001** | |
| **asthma current**^a^  [yes vs. no] | 50/1107  (4.5%) | 88/1088  (8.1%) | **<.001** | | | 72/1122  (6.4%) | 93/1099  (8.5%) | | .075 | |
| **asthma endotype current**^b^ |  |  | **.014** | | |  |  | | .094 | |
| atopic asthma | 31/849  (3.7%) | 57/871  (6.5%) |  | | | 56/1122  (5.0%) | 79/1099  (7.2%) | |  | |
| non-atopic asthma | 11/849  (1.3%) | 16/871  (1.8%) |  | | | 16/1122  (1.4%) | 14/1099  (1.3%) | |  | |
| no asthma | 807/849  (95.1%) | 798/871  (91.6%) |  | | | 1050/1122  (93.6%) | 1006/1099  (91.5%) | |  | |

*p*-values were obtained from Fisher´s exact test for binary variables, chi-squared test for categorical variables with more than two categories and t-test for continuous variables.

Please note that the sample size from asthma current and asthma endotype differ because of specific inclusion criteria: ^a^positive parent-reported medical diagnosis, whistling or wheezing symptoms and medical treatment against asthma (at least two criteria); ^b^specific IgE levels; *%EI: percentage of total daily energy intake.

In contrast to the sex distribution in the main study population, no significant differences in BMI and current asthma between males and females could be detected in the sub-population (endotype-specific asthma group), Table S3. Furthermore, the significant sex-difference in daily sucrose intake (EI%) did not persist within the sub-population. However, the percentage distribution for these variables as well as for the other constructs examined are very similar in both populations.
