# Supplementary material for: Prospective association between psychopathological symptoms in childhood and asthma in adolescence: Results from the GINIplus and LISA birth cohort studies
Source: Pediatr Allergy Immunol. 2025 Jul 24;36(7):e70151. doi: 10.1111/pai.70151 (PMC12287888; doi:10.1111/pai.70151)
Supplement: Supplementary file 4 — Appendix S4. [file PAI-36-e70151-s003.docx]

## Supplement S4. Sensitivity analyses for adjusted^a^ logistic regression and multinomial regression models for the prospective association between SDQ (subscales) at age 10 and asthma at age 15 compared to no asthma at age 15 (as reference) considering FeNO and a positive spirometric bronchodilation response.

Table S4. Significant associations are highlighted in bold.

|  | **current asthma** 15 years  (*n*/*N*=231/3584) | |  | **asthma endotypes** 15 years | | | |
| --- | --- | --- | --- | --- | --- | --- | --- |
|  |  |  |  | **atopic asthma** (*n/N*=135/2221) | | **non-atopic asthma** (*n/N*=30/2221) | |
|  | OR^a^  (95%CI) | *p-value* |  | RRR^a^  (95%CI) | *p-value* | RRR^a^  (95%CI) | *p-value* |
| **SDQ** 10 years  total difficulties^b^ | **2.00**  **(1.15-3.48)** | .015 |  | 1.60  (0.81-3.15) | .177 | **2.94**  **(1.07-8.06)** | .036 |
| **SDQ** 10 years  Emotional problems^b^ | 1.52  (0.86-2.70) | .150 |  | 1.43  (0.71-2.90) | .318 | 1.82  (0.64-5.20) | .266 |
| **SDQ** 10 years  conduct problems^b^ | 0.66  (0.30-1.48) | .314 |  | 0.65  (0.26-1.65) | .368 | 0.38  (0.05-3.03) | .359 |
| **SDQ** 10 years  hyperactivity/inattention^b^ | 1.06  (0.53-2.10) | .878 |  | 0.60  (0.24-1.49) | .273 | 2.49  (0.77-8.06) | .129 |
| **SDQ** 10 years  peer problems^b^ | 1.77  (0.93-3.39) | .082 |  | 2.07  (0.99-4.31) | .053 | 1.91  (0.51-7.19) | .340 |
| **SDQ** 10 years  problems in prosocial behavior^b^ | 1.52  (0.66-3.49) | .324 |  | 1.65  (0.62-4.42) | .319 | 0.70  (0.09-5.71) | .739 |

^a^ covariates and confounders: sex, age (15 years), study group, recruitment region, parental education level, parental atopy, BMI, early-life infections, eczema ever, allergic rhinitis ever, total energy intake (kcal/day), total starch (percentage of total daily energy intake, %EI), total sucrose (%EI), fruits & vegetables (%EI), pubertal status (10 years), **FeNO ≥ 20ppB** (15 years), **positive spirometric bronchodilation response** (15-years)

^b^ [borderline/abnormal vs. normal]

In this sensitivity analyses data were included from participants who voluntarily took part in the on-site fractional exhaled nitric oxide (FeNO) measurement (*N*=2144) and bronchodilation test (*N*=1980) at the 15-year follow-up. FeNO levels were measured using the NIOX MINO® (Areocrine) device, in accordance with established guidelines ^23^. The adolescents were instructed to inhale nitric oxide (NO-)free air to total lung capacity, followed by gradually and consistently exhalation at a constant flow rate (50±5 ml/s). The procedure was repeated until an acceptable value was registered. Based on the cut-off guidelines by the Global Initiative for Asthma (GINA), the obtained NO concentration (parts per billion, ppb) was stratified into low/normal (<20ppB) and high (≥20ppB) ^24^. In the included sample, 37.7% (*n*=809/2144) exhibited values exceeding the cut-off.

The bronchodilator response was performed according to recommendations of the American Thoracic Society/European Respiratory Society (ATS/ERS) ^25^. Following the baseline spirometry, adolescents received Salbutamol as a bronchodilator. 15 minutes after inhalation, the spirometry was repeated. The spirometric indices were obtained from the maneuver with the highest sum of forced expiratory volume in 1 sec (FEV_1_) and forced vital capacity (FVC). A positive spirometric bronchodilation response was defined by an increase of more than 12% and more than 200ml in FEV_1_ and/or FVC from baseline to the post-bronchodilator measurement. This applied to only 4.2% (*n*=84/1980) of the included participants.

The results from the prospective analyses with additional adjustment for FeNO measurement and a positive spirometric bronchodilation response as shown in Table S4 are largely consistent with our primary findings as they also revealed a significant association of psychopathological symptoms at age 10 with asthma at age 15 (OR=2.00, 95%CI=1.15-3.48, *p*=.015), particularly with regard to endotype-specific association with non-atopic asthma (RRR=2.94, 95%CI=1.07-8.06), *p*=.036). Only the observed prospective association between peer problems in childhood and atopic asthma in adolescence did not reach statistical significance, although at least a trend was evident (RRR=2.07, 95%CI=0.99-4.31, *p*=.053).
