# Supplementary material for: Prospective association between psychopathological symptoms in childhood and asthma in adolescence: Results from the GINIplus and LISA birth cohort studies
Source: Pediatr Allergy Immunol. 2025 Jul 24;36(7):e70151. doi: 10.1111/pai.70151 (PMC12287888; doi:10.1111/pai.70151)
Supplement: Supplementary file 5 — Appendix S5. [file PAI-36-e70151-s001.docx]

## Supplement S5. Sex-specific results of adjusted logistic regression and multinomial regression models for the prospective association between SDQ (subscales) at age 10 and asthma at age 15 compared to no asthma at age 15 (as reference).

Tables S5. Significant associations are highlighted in bold.

### a) Adjusted^a^ logistic regression and multinomial regression models for prospective association in females between SDQ at age 10 and asthma at age 15 compared to no asthma at age 15

|  | **females** | | | | | | |
| --- | --- | --- | --- | --- | --- | --- | --- |
|  | **current asthma** 15 years  (*n*/*N*=92/1790) | |  | **asthma endotypes** 15 years | | | |
|  |  |  |  | **atopic asthma** (*n/N*=56/1122) | | **non-atopic asthma** (*n/N*=16/1122) | |
|  | OR^a^  (95%CI) | *p-value* |  | RRR^a^  (95%CI) | *p-value* | RRR^a^  (95%CI) | *p-value* |
| **SDQ** 10 years  total difficulties^b^ | 1.17  (0.50-2.76) | .721 |  | 0.72  (0.20-2.63) | .618 | 1.50  (0.25-8.95) | .655 |
| *new onset of asthma after 10 years (n=44)* | | | |  | | | |
| **SDQ** 10 years  total difficulties^b^ | 1.27  (0.37-4.31) | .704 |  |  |  |  |  |
| *adjusted for asthma 10 years* | | | |  | | | |
| **SDQ** 10 years  total difficulties^b^ | 1.14  (0.41-3.17) | .806 |  |  |  |  |  |

^a^ covariates and confounders: age (15 years), study group, recruitment region, parental education level, parental atopy, BMI, early-life infections, eczema ever, allergic rhinitis ever, total energy intake (kcal/day), total starch (percentage of total daily energy intake, %EI), total sucrose (%EI), fruits & vegetables (%EI), pubertal status (10 years)

^b^ [borderline/abnormal vs. normal]

### b) Adjusted^a^ logistic regression and multinomial regression models for prospective association in males between SDQ at age 10 and asthma at age 15 compared to no asthma at age 15

|  | **males** | | | | | | |
| --- | --- | --- | --- | --- | --- | --- | --- |
|  | **current asthma** 15 years  (*n*/*N*=139/1794) | |  | **asthma endotypes** 15 years | | | |
|  |  |  |  | **atopic asthma** (*n/N*=79/1099) | | **non-atopic asthma** (*n/N*=14/1099) | |
|  | OR^a^  (95%CI) | *p-value* |  | RRR^a^  (95%CI) | *p-value* | RRR^a^  (95%CI) | *p-value* |
| **SDQ** 10 years  total difficulties^b^ | 1.80  (0.97-3.33) | .062 |  | 1.48  (0.67-3.30) | .334 | **5.43**  **(1.30-22.74)** | .021 |
| *new onset of asthma after 10 years (n=46)* | | | |  | | | |
| **SDQ** 10 years  total difficulties^b^ | 1.71  (0.67-4.41) | .264 |  |  |  |  |  |
| *adjusted for asthma 10 years* | | | |  | | | |
| **SDQ** 10 years  total difficulties^b^ | 1.62  (0.75-3.51) | .218 |  |  |  |  |  |

^a^ covariates and confounders: age (15 years), study group, recruitment region, parental education level, parental atopy, BMI, early-life infections, eczema ever, allergic rhinitis ever, total energy intake (kcal/day), total starch (%EI), total sucrose (%EI), fruits & vegetables (%EI), pubertal status (10 years)

^b^ [borderline/abnormal vs. normal]

### c) Adjusted^a^ logistic regression and multinomial regression models for prospective association in females between SDQ subscales at age 10 and asthma at age 15 compared to no asthma at age 15

|  | **females** | | | | | | |
| --- | --- | --- | --- | --- | --- | --- | --- |
|  | **current asthma** 15 years  (*n*/*N*=92/1790) | |  | **asthma endotypes** 15 years | | | |
|  |  |  |  | **atopic asthma** (*n/N*=56/1122) | | **non-atopic asthma** (*n/N*=16/1122) | |
|  | OR^a^  (95%CI) | *p-value* |  | RRR^a^  (95%CI) | *p-value* | RRR^a^  (95%CI) | *p-value* |
| **SDQ** 10 years  emotional problems^b^ | 1.12  (0.53-2.40) | .765 |  | 1.52  (0.53-4.38) | .433 | 0.43  (0.08-2.40) | .333 |
| **SDQ** 10 years  conduct problems^b^ | 0.71  (0.22-2.28) | .567 |  | 0.30  (0.05-1.86) | .197 | <0.010  (<0.010-) | .995 |
| **SDQ** 10 years  hyperactivity/ inattention^b^ | 1.45  (0.49-4.29) | .503 |  | 0.87  (0.18-4.24) | .859 | 1.86  (0.19-18.08) | .595 |
| **SDQ** 10 years  peer problems^b^ | 1.01  (0.36-2.83) | .985 |  | 0.85  (0.12-3.73) | .824 | 3.72  (0.59-23.35) | .161 |
| **SDQ** 10 years  problems in prosocial behavior^b^ | 0.92  (0.24-3.50) | .897 |  | 2.00  (0.38-10.50) | .415 | <0.010  (<0.010-) | .996 |

^a^ covariates and confounders: age (15 years), study group, recruitment region, parental education level, parental atopy, BMI, early-life infections, eczema ever, allergic rhinitis ever, total energy intake (kcal/day), total starch (%EI), total sucrose (%EI), fruits & vegetables (%EI), pubertal status (10 years)

^b^ [borderline/abnormal vs. normal]

### d) Adjusted^a^ logistic regression and multinomial regression models for prospective association in males between SDQ subscales at age 10 and asthma at age 15 compared to no asthma at age 15

|  | **males** | | | | | | |
| --- | --- | --- | --- | --- | --- | --- | --- |
|  | **current asthma** 15 years  (*n*/*N*=139/1794) | |  | **asthma endotypes** 15 years | | | |
|  |  |  |  | **atopic asthma** (*n/N*=79/1099) | | **non-atopic asthma** (*n/N*=14/1099) | |
|  | OR^a^  (95%CI) | *p-value* |  | RRR^a^  (95%CI) | *p-value* | RRR^a^  (95%CI) | *p-value* |
| **SDQ** 10 years  emotional problems^b^ | 1.60  (0.83-3.06) | .158 |  | 1.67  (0.71-3.93) | .245 | **5.78**  **(1.29-25.85)** | .022 |
| **SDQ** 10 years  conduct problems^b^ | 0.90  (0.43-1.89) | .783 |  | 0.78  (0.30-2.02) | .606 | 1.74  (0.30-9.93) | .536 |
| **SDQ** 10 years  hyperactivity/ inattention^b^ | 0.99  (0.50-1.97) | .981 |  | 0.44  (0.15-1.25) | .124 | 2.90  (0.70-12.12) | .144 |
| **SDQ** 10 years  peer problems^b^ | **2.17**  **(1.07-4.41)** | .033 |  | **2.72**  **(1.19-6.23)** | .018 | 1.07  (0.12-9.40) | .952 |
| **SDQ** 10 years  problems in prosocial behavior^b^ | 1.15  (0.50-2.64) | .736 |  | 1.16  (0.39-3.45) | .784 | 1.96  (0.20-19.04) | .563 |

^a^ covariates and confounders: age (15 years), study group, recruitment region, parental education level, parental atopy, BMI, early-life infections, eczema ever, allergic rhinitis ever, total energy intake (kcal/day), total starch (%EI), total sucrose (%EI), fruits & vegetables (%EI), pubertal status (10 years)

^b^ [borderline/abnormal vs. normal]

Our findings of the primary analyses were largely replicable for males (Tables S5b and S5d). Hence, a trend was observed in the prospective association between psychopathological symptoms at age 10 and asthma at age 15 (OR=1.80, 95%CI=0.97-3.33, *p*=.062), although the result did not reach statistical significance. The associations between psychopathological symptoms and non-atopic asthma (RRR=5.43, 95%CI=1.30-22.74, *p*=.021), as well as between peer problems and atopic asthma (RRR=2.72, 95%CI=1.19-6.23, *p*=.018), remained stable. Furthermore, males with emotional problems in childhood showed an increased occurrence of non-atopic asthma in adolescence (RRR=5.78, 95%CI=1.29-25.85, *p*=.022) compared to those without such difficulties. In females, no significant effects of psychopathological symptoms in childhood on asthma in general or asthma endotypes in adolescence were observed (Tables S5a and S5c). These findings could indicate a potential influence of sex, even in the absence of statistical significant interaction terms. Again, it is important to consider that the wide range of some CIs potentially impact the statistical significance.
