# Supplementary material for: Prospective association between psychopathological symptoms in childhood and asthma in adolescence: Results from the GINIplus and LISA birth cohort studies
Source: Pediatr Allergy Immunol. 2025 Jul 24;36(7):e70151. doi: 10.1111/pai.70151 (PMC12287888; doi:10.1111/pai.70151)
Supplement: Supplementary file 6 — Appendix S6. [file PAI-36-e70151-s004.docx]

**Appendix S6.**

GINIplus study group comprises the Helmholtz Zentrum München, German Research Centre for Environmental Health, Munich (Heinrich J., Wichmann H.E., Sausenthaler S., Zutavern A., Chen, C.M., Schnappinger M., Rzehak P.); Research Institute, Department of Pediatrics, Marien-Hospital Wesel (Berdel D., von Berg A., Beckmann C., Groß I.); Department of Child and Adolescent Psychiatry, Psychosomatics and Psychotherapy, Hospital of the Ludwig-Maximilians-University (LMU) Munich (Koletzko S., Reinhardt D., Krauss-Etschmann S.); Department of Pediatrics, Technical University of Munich, Munich (Bauer C.P., Brockow I., Grübl A., Hoffmann U.); IUF – Leibniz-Research Institute for Environmental Medicine at the University of Düsseldorf (Krämer U., Link E., Cramer C.); Centre for Allergy and Environment, Technical University, Munich (Behrendt H.).

LISA study group comprises the Helmholtz Zentrum München, German Research Centre for Environmental Health, Munich (Heinrich J., Wichmann H.E., Sausenthaler S., Chen C.M., Schnappinger M.); Department of Pediatrics, Municipal Hospital “St.Georg”, Leipzig (Borte M., Diez U.); Research Institute, Department of Pediatrics, Marien-Hospital Wesel (von Berg A., Beckmann C., Groß I.); Pediatric Practice, Bad Honnef (Schaaf B.); Helmholtz Centre for Environmental Research – UFZ, Department of Environmental Immunology/Core Facility Studies, Leipzig (Lehmann I., Bauer M., Gräbsch C., Röder S., Schilde M.); University of Leipzig, Institute of Hygiene and Environmental Medicine, Leipzig (Herbarth O., Dick C., Magnus J.); IUF – Leibniz-Research Institute for Environmental Medicine at the University of Düsseldorf (Krämer U., Link E., Cramer C.); Department of Pediatrics, Technical University of Munich, Munich (Bauer C.P., Brockow I., Hoffmann U.); ZAUM - Center for Allergy and Environment, Technical University, Munich (Behrendt H., Grosch J., Martin F.).
